# Land-use/cover conversion affects soil organic-carbon stocks: A case study along the main channel of the Tarim River, China

**DOI:** 10.1371/journal.pone.0206903

**Published:** 2018-11-15

**Authors:** Yuhai Yang, Yaning Chen, Zhi Li, Yapeng Chen

**Affiliations:** State Key Laboratory of Desert and Oasis Ecology, Xinjiang Institute of Ecology and Geography, Chinese Academy of Sciences, Urumqi, Xinjiang, China; Pacific Northwest National Laboratory, UNITED STATES

## Abstract

Soil organic carbon (SOC) constitutes a large pool within the global carbon cycle. Changes in land-use/cover strongly drive variation of SOC stocks. We analyzed the changes in four types of land use/cover and their influence on SOC content, density, and regional stocks along the main channel of the Tarim River in China for 2000–2010 obtained from remotely sensed images and field surveys. The areas and structures of the land uses/covers changed greatly during this period. Specifically, the areas of cultivated, industrial and residential, and shrub land increased, particularly cultivated and shrub land. The areas of forestland, grassland, water bodies, and unused land decreased. SOC stocks in forestland, grassland and unused land decreased between 2000 and 2010. The total SOC stock for the forestland shrub land grassland and unused land was lower in 2010 than 2000. Land-use/cover conversion thus affected SOC stocks. Specifically, conversions from forestland to shrub land, forestland to grassland, forestland to unused land, grassland to shrub land, grassland to unused land, and shrub land to unused land decreased the SOC stocks. This study provides a scientific basis for eco-environmental protection in arid areas.

## Introduction

Soil carbon is the largest pool of carbon in the terrestrial biosphere and has thus received considerable attention in recent decades. Its interactions with atmospheric and biospheric carbon pools make it a critical component of the global carbon cycle [1]. Whether soils act as carbon sinks or sources has become a major focus of research on global climate change [2]. Soil contains the largest pool of organic carbon, approximately 615 Pg C at a depth of 0.2 m, 1550 Pg C at 1 m, and 2344 Pg C at 3 m, in global terrestrial ecosystems [3]. The global pool of soil organic carbon (SOC) has been estimated at >3.3-fold larger than the atmospheric carbon pool and 4.5-fold larger than the biotic pool [4]. The SOC pool, however, is susceptible to human interference, primarily changes in land use/cover. Land-use/cover changes represent major anthropogenic contributions to emissions of greenhouse gases [5,6]. Changes in land use have important effects on regional ecological processes and global climate change [7] and have been associated with approximately 20% of the global CO_2_ emissions to the atmosphere [8,9]. Land use affects SOC concentrations [10] and stocks [11,12], and land-use/cover conversion influences natural forestland, farmland, cropland, and shrub land to a depth of 30 cm. Conversion of forests to agricultural ecosystems negatively affects SOC concentration and stock by 20–50% [13,14]. Most studies have been conducted in tropical or subtropical areas, because these regions are highly productive and have dense carbon stocks [15], but the results have been variable [16]. Quantification and continuous assessment of changes in carbon pool sizes and fluxes are fundamental to elucidating the effects of changes in land use/cover on ecosystemic functioning and to limiting the emissions of greenhouse gases [17]. The issue of land-use/cover change is especially critical in China, where land resources per capita are well below the global average [18,19]. Comprehensive studies of SOC content and storage associated with land-use/cover change, however, remain rare in temperate arid regions.

The Tarim River is one of the longest inland rivers in the world. Its basin is the largest inland river basins and a major production area of grain and cotton in China. The basin also contains abundant natural resources but is ecologically fragile [20]. Land use/cover in the basin has been complexly changed and converted in recent decades, due to intense human disturbance and regional climate change [21]. The basin is the largest habitat for *Populus euphratica* in the world, which shrank from 454000 ha in the 1950s to 247300 ha in 2000 [22]. Most studies in the basin have focused on ecosystemic degradation, water stress, changes in area of land uses/covers and rates of change in various periods [23,24]. Few studies, though, have addressed the influence of land-use/cover change on SOC stocks along the main channel of the Tarim River. Our recent works indicated that the area change influenced soil organic carbon storage for the same land use/cover type which was applied on the discussion of changes on the organic carbon storage in the main stream of Tarim River [25]. However, the influence of land area transformation among different land use/cover types on soil organic carbon storage, especially the influence for unequal transition area among the different land use/cover types on regional soil organic carbon storage has not been done. Such studies are thus profoundly needed, given the former and current high levels of land cover change along the main channel. Understanding the changes in SOC stocks caused by changes in land-use/cover in the basin is therefore essential, especially in the context of climate change and the potential sink of soil organic matter for atmospheric CO_2_.

The objectives of the current study are to: (i) evaluate land-use/cover changes, and (ii) determine the influence of land-use/cover conversion on SOC stock from 2000–2010 along the main channel of the Tarim River. The current study uses the same dataset [25], including the research land area, the research period of land use /cover, area of different land use/cover types, the background value of soil characteristic parameters in the research land, and calculation method of soil organic carbon storage, but presents some additional analyses. Such quantitative analyses will provide insight for the management of land use and will constitute a scientific basis for eco-environmental protection in arid areas.

## Materials and methods

### Study area

The Tarim River Basin in northwestern China, mainly between 34–45°N and 73–97°E, is the largest inland river basin in China, with an area of 1020000 km^2^. Most of the Tarim River traverses the Taklamakan Desert, one of the world's largest desert. The main channel of the river, which is defined as the river course below the confluence of the Hetian, Yarkan, and Akesu Rivers, is 1320 km in length and covers an area of 17600 km^2^. Our study area was bounded in the north by the lower edge of an inclined plain along the southern slopes of the Tianshan Mountains and in the south by the edge of the Taklamakan Desert. The region has a typical continental climate: arid, with precipitation usually <50 mm and evaporation as high as 2300–3000 mm; abundant sunlight, with annual sunshine durations of 2800–3100 h; frost-free period of 185–210 d; mean annual temperature of 10–11°C; and accumulated temperature ≥10°C up to 4000–4350°C. The area belongs to a warm temperate zone with sparse shrubs and subshrub desert. The alluvial plain of the Tarim River has been built up by thick quaternary deposits consisting of fine sand in the upper layer and clay and silt in the deeper layer. The landscape comprises a desert riparian forest on a flat floodplain with slopes ≤3%. The major plant species mainly belong to the families Salicaceae, Tamaricaceae, Leguminosae, Apocynaceae, and Gramineae. The species include trees such as *P*. *euphratica*; shrubs such as *Tamarix* spp., *Lyciumruthenicum*, and *Halimodendron halodendron*; and herbaceous plants such as *Phragmites australis* (Cav.) Trin. ex Steud, *Poacynum hendersonii*, *Alhagi sparsifolia*, and *Karelinia caspica*. The zonal soil is a brown desert soil, and the area of solonchak soil is large. Meadow soil and aeolian sandy soil are also distributed in some areas [26].

### Data collection

Data for land use/cover were derived from Landsat Thematic Mapper images, including Landsat bands 5, 4, and 3 for 8 August 2000 and 9 August 2010. These images were selected for their good quality (i.e. they contained few clouds). We also referenced the classification system of land use/cover for arid regions in China, which includes primary and secondary categories, where classification of the secondary category is the breakdown based on the primary category. The classification system for the primary category includes six types: water body (WB), forestland (FL), grassland (GL), cultivated land (CL), residential and industrial land (RL), and unused land (UL) [27]. The classification system for the secondary category includes WB, FL, shrub land (SL), sparsely forestland, low-coverage grassland, moderate-coverage grassland, high-coverage grassland, CL, RL, and UL. The classification used ENVI and ArcGIS software, augmented by GPS on field trips [27,28]. The FL site in this study does not contain SL; the GL site includes low-, moderate-, and high-coverage grassland; and the UL site is bare land.

### Sampling and laboratory analysis

**Fig 1:**
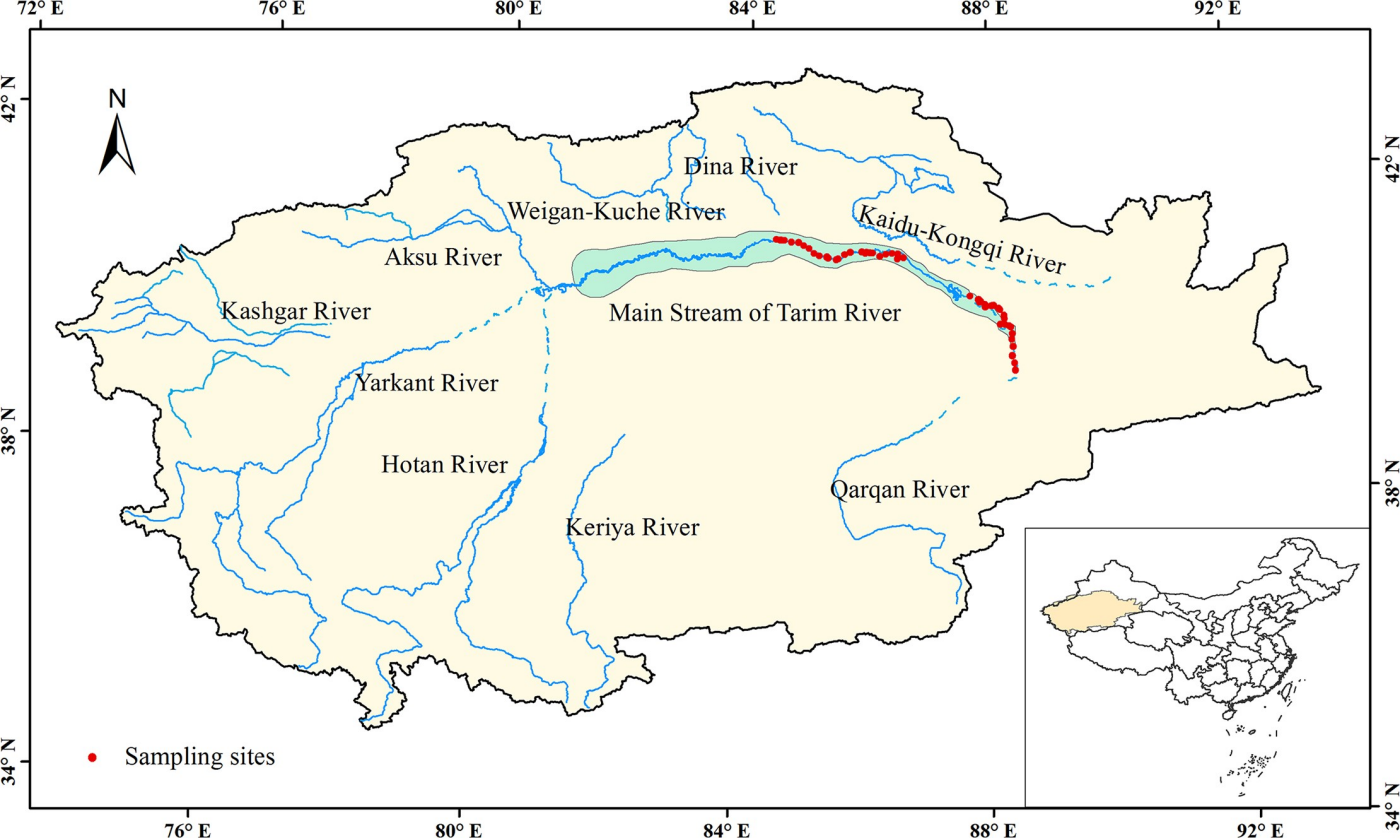
Location map of the Tarim River basin, Xinjiang, China.

We chose FL, SL, GL, and UL, which represent the main land uses/covers in this area, for analyzing the influence of their changes on SOC stocks during 2000–2010. We chose a typical natural forest containing mainly *P*. *euphratica* to represent FL, a typical single *Tamarisk* community to represent SL, a *Phragmites australis* (Cav.) Trin. ex Steud community to represent GL, and bare land to represent UL to reduce the influence of human disturbances such as fertilization. Soil samples were collected in September 2010 in the middle and lower reaches of the study area along the Tarim River (Fig 1). The four selected sampling sites had been minimally disturbed by human activities and animal grazing. The upper reaches of the Tarim River were not sampled due to poor accessibility. Soil samples were collected to a nominative depth of 1m from pits (150 cm long × 70 cm wide) dug for soil collection. Samples were collected from six layers (0–5, 5–15, 15–30, 30–50, 50–80, and 80–100 cm) at each site. Sampling activites don't require specific permissions because the study area isn't a national nature reserve. Moreover, the collected soil samples were used only for scientific research. The field studies did not involve endangered or protected species. A total of 56 samples were collected, with 30, 10, 8, and 8 from FL, SL, GL, and UL, respectively. Each sample was a composite from five points within the same layer. Roots were removed from the samples, which were then air-dried at room temperature and passed through a 2-mm sieve. Coarse gravel (>2 mm) which does not contain organic matter were removed, and their percentage was calculated for each layer by oven-drying at 67°C for 24 h. The organic-matter content was subsequently determined (K_2_-Cr_2_O_7_-H_2_SO_4_ Walkley-Black oxidation; Laboratory of Soil Physics, 1978) [29], which was multiplied by a correction factor of 0.58 to obtain the SOC content [30].

Soil bulk density of the various layers at the sampling sites was measured using a bulk soil sampler with a stainless-steel cutting ring 5.0 cm in diameter and 5.0 cm long (three replicates) at points adjacent to the sampling points. It was calculated by measuring the original volume of each core and the dry mass after oven-drying at 105°C, which enabled the estimation of the mass of carbon.

### Calculations

We selected SOC density (*D*_*soc*_) and regional SOC storage (*S*_*soc*_) to analyze the storage of SOC in the four land uses/covers.

*D*_*soc*_ refers to the SOC reserve per unit area in a soil horizon at a specific depth, which is generally 1 m. Not all soil horizons, however, reach a depth of 1 m or more due to soil developmental differences. *D*_*soc*_ (kg·m^-2^) of each horizon can be calculated by Eq 1 [31]:

$$D_{soci}=S_{\mathrm{oci}}\times H_{i}\times {\mathrm{BD}}_{i}\times {(100‑\mathrm{CFW}}_{i})/100$$ (1)

where i denotes the 0–5, 5–15, 15–30, 30–50, 50–80, or 80–100 cm layer; and H_i_, S_oci_, BD_i_, and CFW_i_ are the layer interval (cm), SOC content (g·kg^-1^), soil bulk density (g·cm^-3^), and percentage of coarse gravel (>2 mm) by weight [32] of horizon i of the soil profile, respectively. If a soil profile has more than one layer, *D*_*soc*_ (kg·m^-2^) can be calculated by:

$$Dsoc=\sum_{i=1}^{n}S_{\mathrm{oci}}\times H_{i}\times {\mathrm{BD}}_{i}\times (100‑{\mathrm{CFW}}_{i})/100$$ (2)

where n is the number of layers [27–29]. We calculated *D*_*soci*_ for each layer using Eq 1 and calculated *D*_*soc*_ for the 0–100 cm profile (*D*_*soc100*_) using Eq 2 for the four types of land use/cover. To further compare SOC storage, we set 5 cm as the standard depth for calculating *D*_*soci*_ (*D*_*soc5*_) for each layer by:

$$D_{soc5}=S_{\mathrm{oci}}\times 5\times {\mathrm{BD}}_{i}\times (100‑{\mathrm{CFW}}_{i})/100$$ (3)

where 5 is the layer interval (cm).

To calculate *D*_*soci*,_ *D*_*soc100*_ and *D*_*soc5*,_ CFW_i_ is estimated by Eq 4:

$$CFW_{i}\left(\%\right)=\left(\mathrm{weight}\;\mathrm{not}\;\mathrm{passing}\;a\;2‑\mathrm{mm}\;\mathrm{sieve}\right)\times 100/\mathrm{total}\;\mathrm{weight}\;\mathrm{of}\;\mathrm{soil}$$ (4)

To account for the contribution of *D*_*soci*_ of different soil layers to *D*_*soc*_ for the 0–100 cm profile, the following formula (Eq 5) was applied.

$$PD_{i}/PD_{100}=D_{soci}\times 100/D_{soc100}$$ (5)

where *PD*_*i*_*/PD*_*100*_ is the proportion of *D*_*soci*_ of layer i compared to *D*_*soc*_ for the 0–100 cm profile.

*S*_*soc*_ refers to the total SOC stock within the region. To assess the total SOC stock of unique area awith specific land use/cover and total SOC stock of the entire study area, the following formula (Eq 6) was applied:

$$S_{soc}=A\times D_{soc}$$ (6)

where A is land area (m^2^). We estimated *S*_*soc*_ for 2000 using data based on land area in 2000 and *D*_*soc100*_ of 2010, and estimated S_*soc*_ for 2010 using data based on area in 2010 and *D*_*soc100*_ of 2010 in the four land uses/covers by Eq 6, respectively.

In this study, *D*_*soci*,_ *D*_*soc100*,_ *D*_*soc5*_ and PD_i_/PD_100_ are used to analyze SOC density. S_soc_ is used to analyze regional SOC storage. We used an index of change in magnitude of land use/cover(*Li*) (km^2^/(100 km^2^/a) defined as a change of unit area of land use/cover j in the study period (a, b) in region or space i [33] to reflect the change of land use/cover in time scale. The index was calculated as:

$$Li=(K_{j,b}‑K_{j,a})/L_{\mathrm{Ai}}/T$$ (7)

where K_j,a_ and K_j,b_ are the areas (hm^2^) of land use j in area i in periods a and b, respectively, L_Ai_ is the area (hm^2^) of region i, and T is the time interval in periods a and b, a.

### Statistical analysis

A one-way ANOVA was used to detect significant differences in SOC contents and soil bulk densities among the four land uses/covers, and multiple comparisons were conducted using the least standard difference test when the variances were homogeneous. Differences were considered significant at *P*<0.05. Primary statistical analyses were conducted with SPSS 13.0 (SPSS Inc., Chicago, USA). Bar graphics were produced using SigmaPlot 12.0 (Systat Software Inc., San Jose, USA).

In this study, a general overview on the land use/cover changes is provided at first, and all land use/cover types are taken into consideration in this section. Secondly, only FL, SL, GL, and UL were selected to analyze SOC content, soil bulk density, and SOC density in 2010. Finally, only the four land/cover types are analyzed in regional SOC stocks in 2000 and 2010, and the influence of four land use/cover types conversions on SOC stocks during 2000–2010.

## Results

### Land-use/cover change

**Fig 2:**
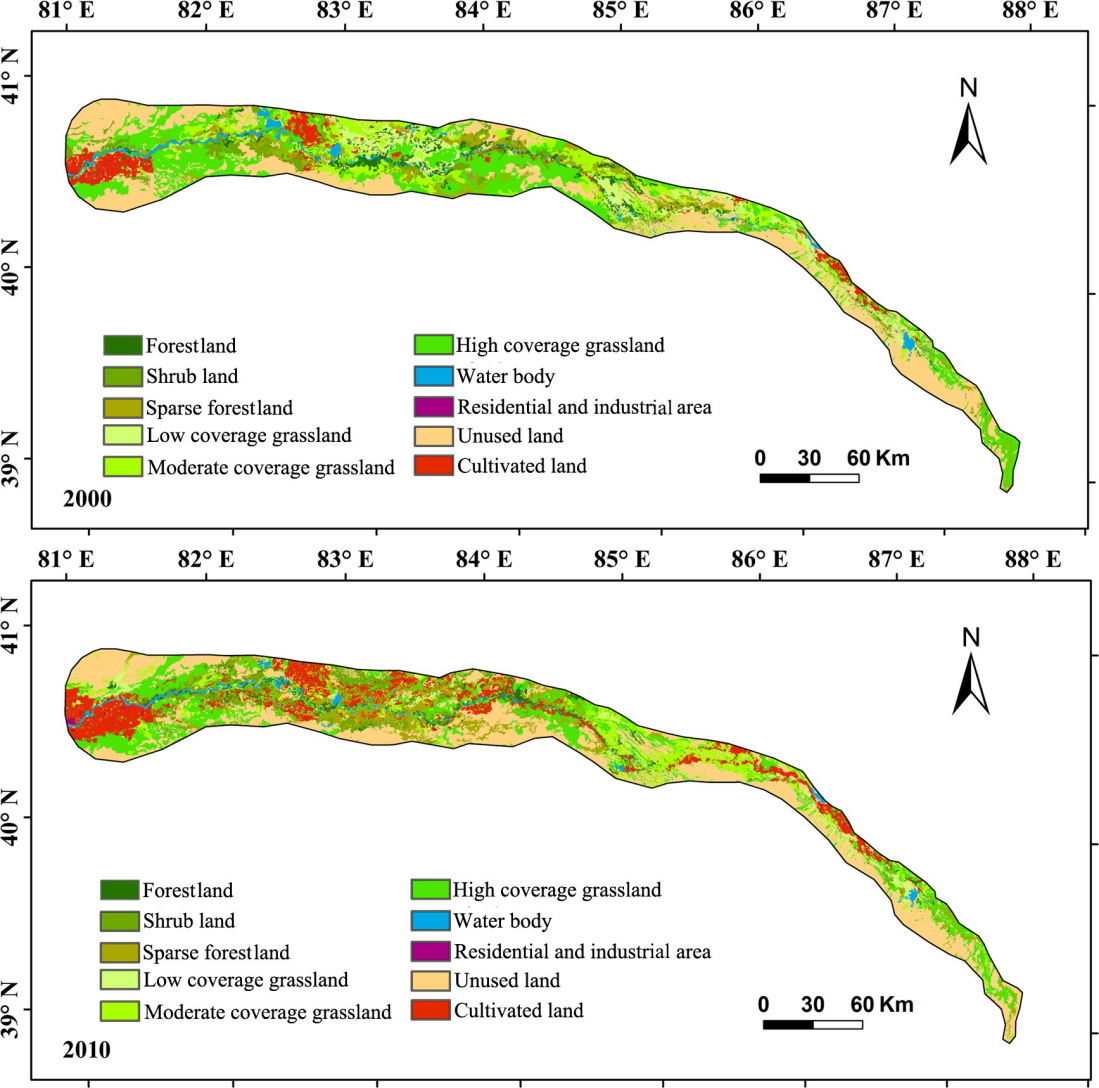
Land use/cover change along the main channel of the Tarim River (the data are adapted from [25]).

**Fig 3:**
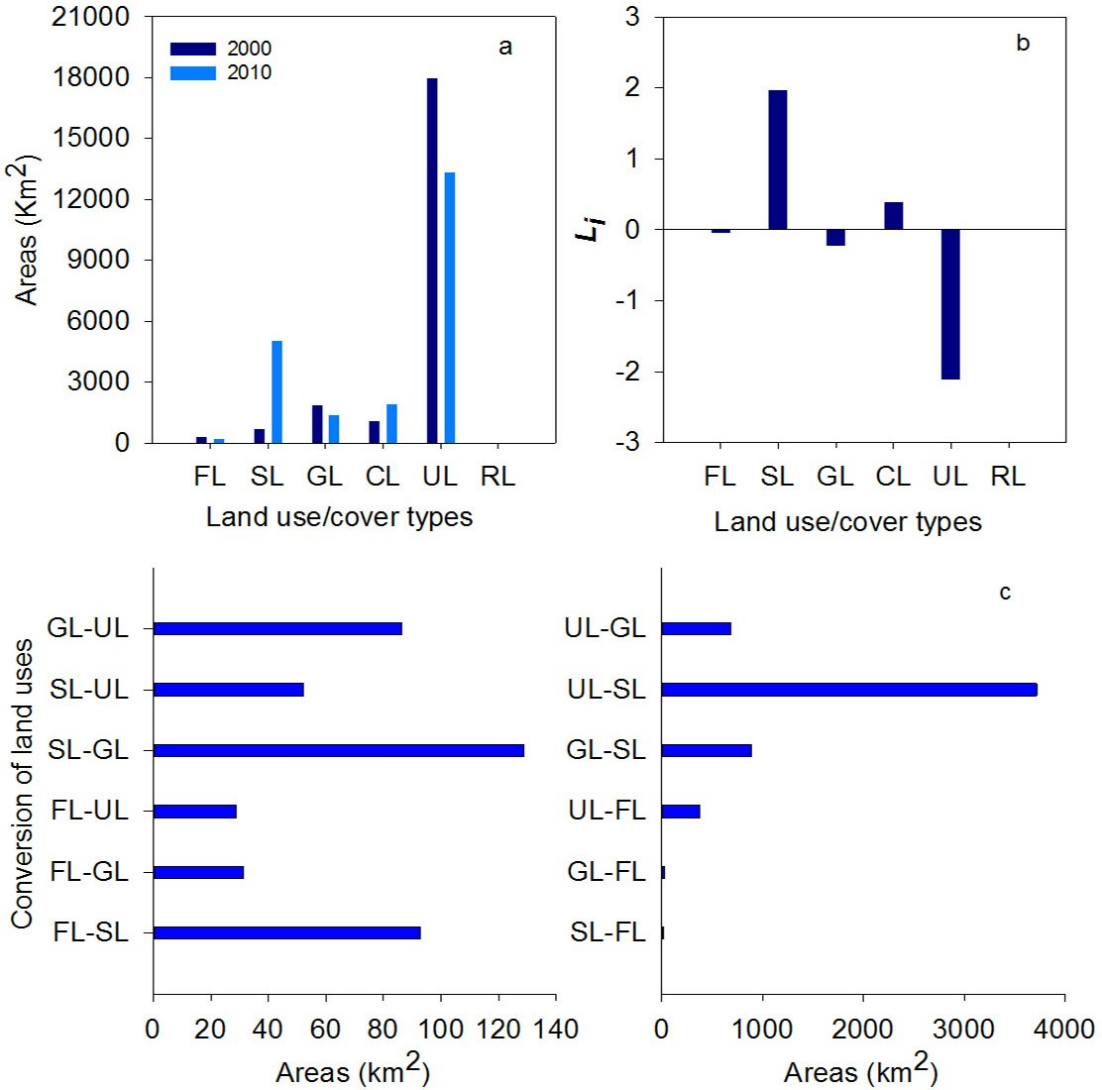
Area changes of different land use/cover and theirs conversion (the data are adapted from [25]).

The area and structure of land use/cover changed considerably between 2000 and 2010. The areas of CL, RL, and SL have increased since 2000, particularly CL and SL. The areas of FL (including sparsely forestland), GL, WB, and UL have decreased since 2000, especially FL by 47.65% (Fig 2). CL has greatly expanded since 2000 at 85.97 km^2^·y^-1^, increasing the area by 80%. The area of FL decreased the fastest, by 100.09 km^2^ within 10 years. The structures of the land-use/cover types were mutually converted over the decade, and areas differed among the conversions (Fig 3A). *Li* indicated that UL changed greatly in area, and RL changed only slightly in area (Fig 3B). Specifically, the areas of conversion were greater for FL-SL than SL-FL, similar between FL-GL and GL-FL, less for FL-UL than UL-FL, less for SL-GL than GL-SL, much greater for UL-SL than SL-UL, and much less for GL-UL than UL-GL. One land-use/cover type tended to be converted to multiple types, such as UL substantially converting to SL, GL, and FL during 2000–2010 (Fig 3C).

### Soil organic carbon content and soil bulk density in 2010

**Fig 4:**
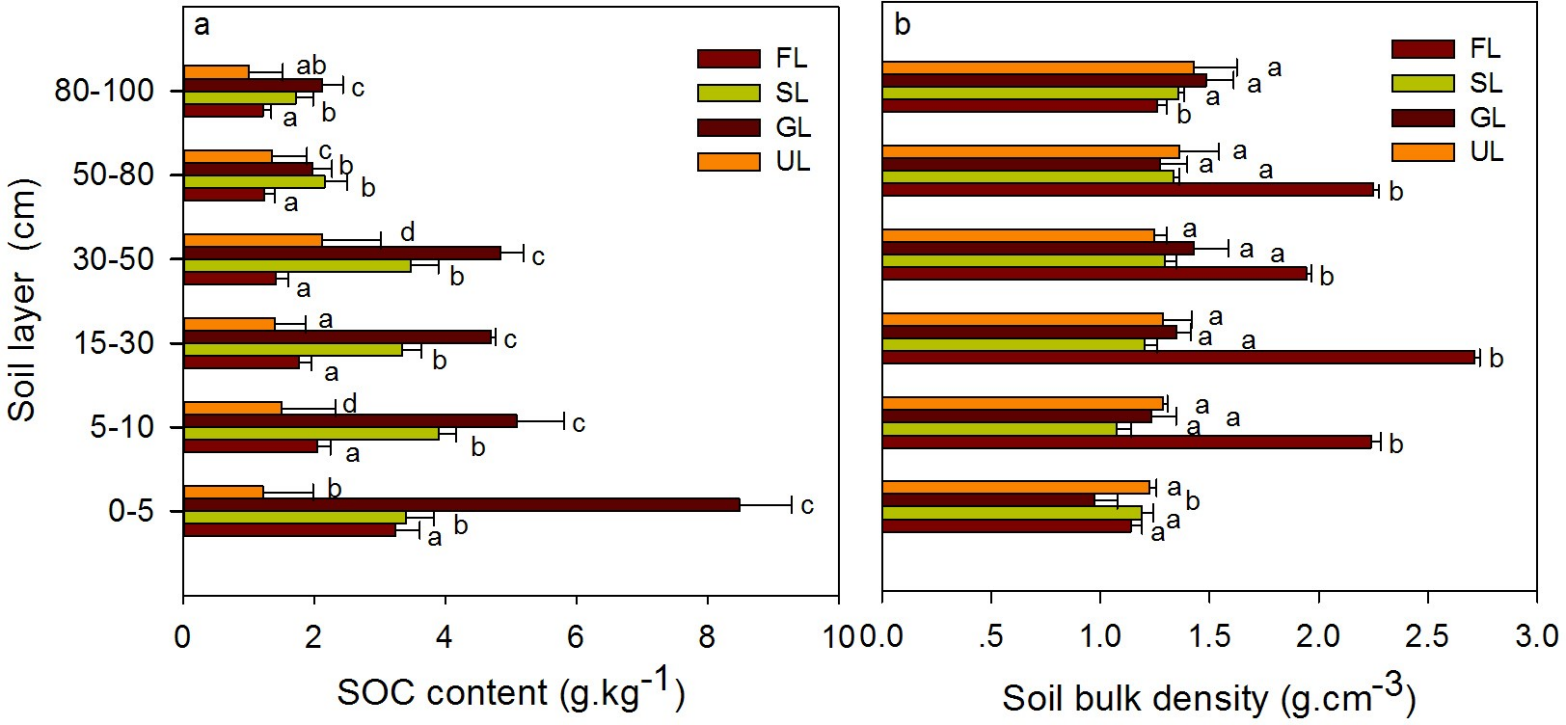
SOC content and soil bulk density in the four land use/cover types in 2010. Bars and error bars represent mean and standard errors, respectively. Different letters above the bars show significant differences (P<0.05) among four land use/cover types.

SOC content decreased with depth except for UL in 2010. The sequence of SOC content in the 30–50 and 50–80 cm layers was GL>SL>UL>FL, and the content differed significantly (P<0.05) among the four land uses/covers, except between SL and GL in the 50–80 cm layer. The sequence of SOC content in the 0–5, 5–15, 15–30, and 80–10 cm layers was GL>SL>FL>UL, and the content varied among the four land uses/covers. Soil bulk density was lowest in the topsoil layer (0–5 cm) in all four types and decreased with depth in GL, SL, and UL (Fig 4A). Soil bulk density in the 80–100 cm layer differed significantly (P<0.05) between FL and the other types but did not differ significantly among SL,GL, and UL. Soil bulk density in the other layers differed significantly (P<0.05) between GL and the other types but did not differ significantly among FL, SL, and UL (Fig 4B).

### Soil organic carbon density in 2010

**Fig 5:**
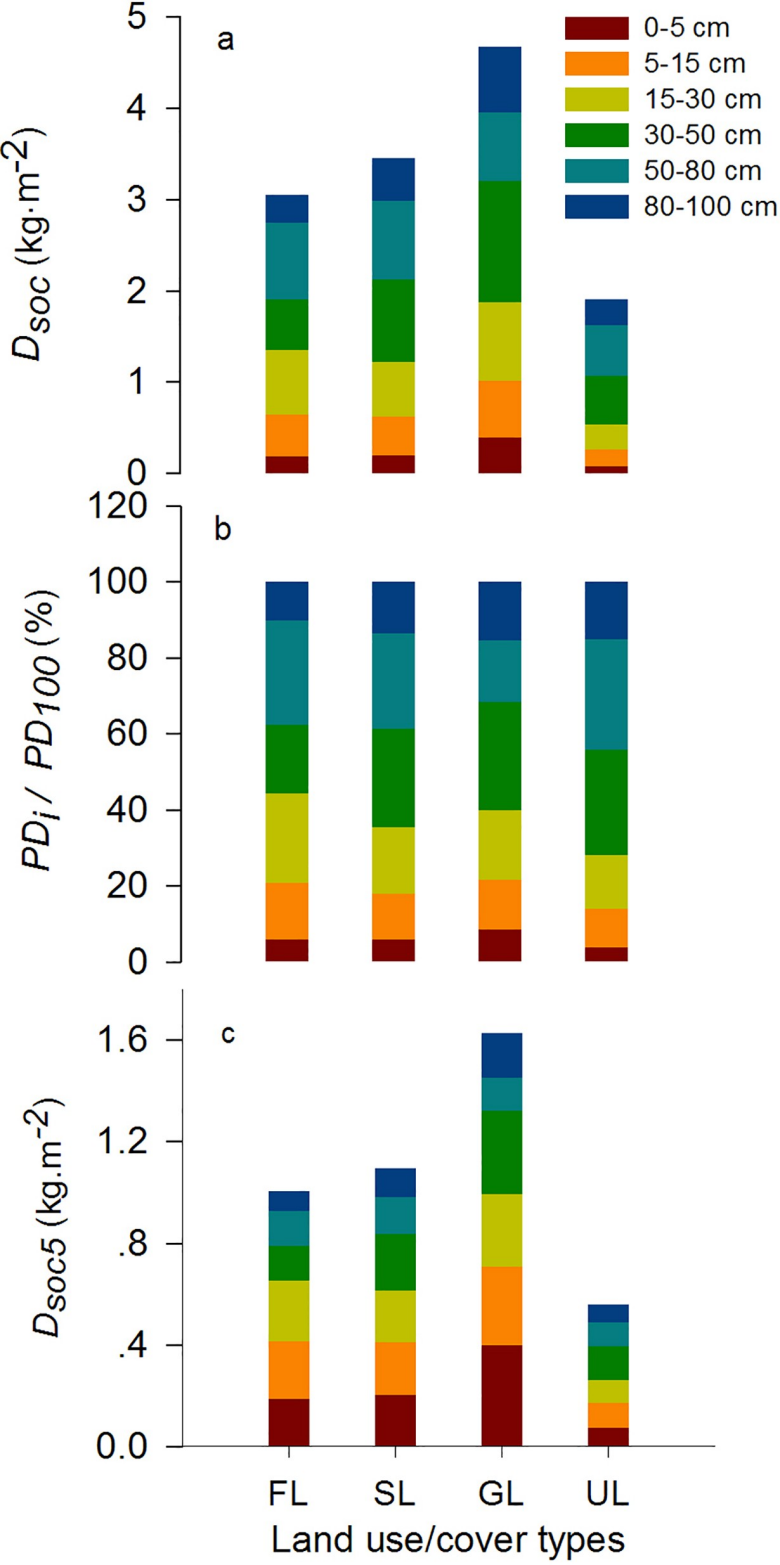
Vertical distribution of *D*_*soc*_ in the four land use/cover types in 2010.

The vertical distribution of *D*_*soc*_ differed among the four types of land use/cover, and *D*_*soc100*_ was highest in GL and lowest in UL (Fig 5A) in 2010. Specifically, *D*_*soc*_ was highest in the 30–50 cm layer for GL and SL and in the 50–80 cm layer for FL and UL. *PD*_*i*_*/PD*_*100*_ can indicate the vertical distribution of *D*_*soc*_. The sequences of *PD*_*i*_*/PD*_*100*_ were GL>SL>FL>UL for the 0–5 cm layer, FL>GL>SL>UL for the 5–15 and 15–30 cm layers, SL≈GL>FL>UL for the 30–50 cm layer, UL≈FL>SL>UL for the 50–80 cm layer, and GL≈UL>SL>FL for the 80–100 cm layer (Fig 5B). the sequences of *D*_*soc5*_ were GL>SL>FL>UL for the 0–5 cm layer, GL>FL>SL>UL for the 5–15 and 15–30 cm layers, GL>SL>UL≈FL for the 30–50 cm layer, SL≈FL>GL>UL for the 50–80 cm layer, and GL>SL>FL≈UL for the 80–100 cm layer (Fig 5C).

### Regional soil organic carbon stocks in 2000 and 2010

*S*_*soc*_ for the 0–100 cm profiles had decreased in three of the four land-use/cover types but had increased in SL by 2010. *S*_*soc*_ for the 0–100 cm profile in SL in 2010 is 7 times as much as that in 2000, and in FL, GL and UL it is about 0.7 times as much as those in 2000. Total *S*_*soc*_ in the four land use/cover types in 2000 was 130.32×10^7^ kg more than that in 2010. The change in *S*_*soc*_ was largely due to variation in area. Compared with 2000, only the area of SL increased in 2010 among the four land use/cover types. However, if variability of SOC content and soil bulk density over time are taking into consideration, the *S*_*soc*_ change in the four land use/cover types needs further study.

### Conversion of land use/cover affects soil organic carbon stocks

**Fig 6:**
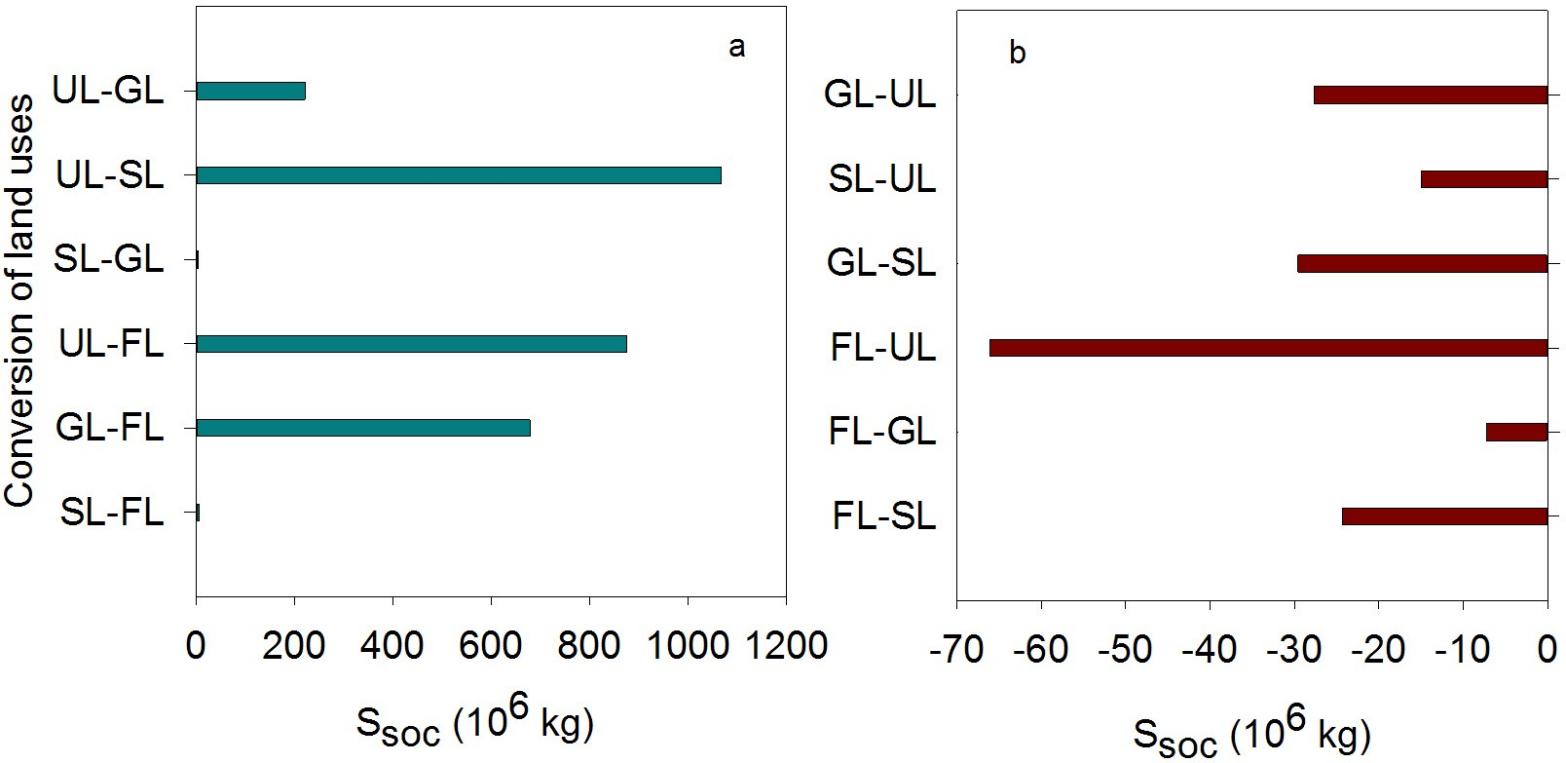
*S*_*soc*_ changes in different conversions of the four land use/cover types (the data are adapted from [25]).

The differences in soil bulk density, *D*_*soc*_, and area indicated that land-use/cover conversion affected *S*_*soc*_ in the 0–100 cm profiles. Specifically, *S*_*soc*_ decreased in the FL-SL, FL-GL, FL-UL, GL-SL, GL-UL, and SL-UL conversions. These conversions represent reverse ecosystem succession. *S*_*soc*_ increased for the opposite conversions in the sequence UL-SL>UL-FL>GL-FL>UL-GL>SL-FL≈SL-GL. These conversions represent positive ecosystem succession. The amount of increased *S*_*soc*_ in the UL-SL is about 248 times as much as that in the SL-GL, and is about 475.02×10^6^ kg higher than average value for these positive conversions. Increased *S*_*soc*_ in the all positive conversions are 3020.35×10^6^ kg more than that in the all opposite conversions. The areas of conversion were similar for FL-GL and GL-FL, but the increase in *S*_*soc*_ was obviously lower for GL-FL than FL-GL (Fig 6A and 6B). The area of conversion was obviously lower for SL-FL than SL-GL, but the increase in *S*_*soc*_ in the 0–100 cm profiles was similar between SL-FL and SL-GL, demonstrating that the conversion of SL to FL was more advantageous for increasing *S*_*soc*_.

## Discussion

Most studies of the effect of land-use/cover change on SOC have focused on the topsoil layer (0–20), which contains the highest levels of SOC and the greatest microbial activity [34,35]. Various studies have also reported inconsistent results for the influence of land-use/cover change on SOC [36–38]. SOC content decreased by 20–43% after natural forest or perennial grassland was converted to agricultural land [39]. Changes in land use in the form of a conversion of forest to grassland and/or cropland induces large changes in SOC dynamics [40], leading to carbon losses of 10–55% [39,41]. SOC content decreased by 20 and 40% within five years after forestland was converted to pasture or farmland, respectively [42]. The SOC contents of FL, SL, and GL were higher in the 0–5, 5–15, 15–30, and 30–50 cm layers than the 50–80 and 80–100 cm layers, indicating that studies of the effect of land-use/cover change on SOC should focus on deep soil layers (>20). The SOC content was highest in GL, perhaps due to the higher root biomass and residues in grasslands. High root biomass and residues can potentially provide more organic matter as the carbon source and can aid the retention of water in soil, which creates favorable conditions for the decomposition of organic matter [43].

Conversions from natural woody vegetation to grassland or barren land are generally accompanied by large changes in soil bulk density. Conversion from FL to UL in our study, however, was not accompanied by large changes in soil bulk density in the 5–100 cm layers. Soil bulk density in these layers did not differ significantly among SL, GL, and UL, and the soil bulk density in the of 80–100 cm layer was significantly lower in FL than the other land uses/covers, likely because root material accumulated in the deep soil of the *P*. *euphratica* forest and because the densities of fine-root surfaces and lengths increased with depth [44].

SOC stocks of the four land uses/covers for 2000 were based on SOC content and soil bulk density in 2010, which may over- or underestimate *S*_*soc*_ for 2000, because SOC content and soil bulk density may change with variations in vegetation growth over time, even within the same type of land use/cover. For example, SOC content was 14.8% lower for cultivated than uncultivated grassland dominated by *Stipa baicalensis* in a typical temperate grassland in Inner Mongolia [45]. Long-term monitoring of SOC content and soil bulk density at the same sites is therefore critical for estimating SOC stocks over a long period. We did not examine the influence of conversion on SOC stocks between CL and the four types of land use/cover, because SOC for CL was severely affected by crop species, fertilization, plowing, irrigation, and reclamation time [43]. Cultivation usually markedly decreases soil carbon [46]. The conversion of farmland to FL can have a positive, negative, or no influence on SOC. For example, conversion to FL did not obviously affect SOC accumulation in the 0–100 cm profiles within 10 years but obviously increased it over 28 years in a *Robinia pseudoacacia* forest in the Loss hilly region of China [47].

The capacity of soil carbon stocks depends on abiotic factors such as mineralogical composition and climate and on soil management and use [48,49]. *D*_*soc*_ in the 0–100 cm profiles in our study differed among the four land-use/cover types and varied with depth. These results demonstrated that the land-use/cover changes affected *D*_*soc*_, which led to changes of *S*_*soc*_. *D*_*soc*_ in the 0–100 cm layers decreased by 35% when the natural secondary forestland was converted to farmland and particularly decreased in the 0–50 cm layers by 20–79% in the farmland. *D*_*soc*_ in the 0–30 cm layers was obviously lower in the desert riparian meadow than in alpine meadows, alpine grasslands, and alpine shrub meadows [50]. *D*_*soc*_ was also below average for surface soils in China [51], indicating that the area of main plant communities and successional change will affect SOC storage in riparian forests, which may affect the global climate. Moreover, this study only considered the changes in area of the land-use/cover types between 2000 and 2010 but not the diversity within the same type or the progress of succession when analyzing *S*_*soc*_.

UL is likely to be severely or mildly desertified, and FL may be in a state of degradation, which may affect the estimates of *S*_*soc*_ when considering the influence of land-use/cover conversion on SOC. For example, stocks of sandy SOC decreased by 90.1% between potential and severe desertification and by 52.2, 49.5, 46.2, and 24.0% in the successions from potential to mild degradation, mild to moderate degradation, moderate to severe degradation, and severe to serious degradation, respectively [44]. The area and degree of desertification continuously increased in the lower reaches of the Tarim River, mainly in areas of very serious desertification, which increased dramatically from1959 to 1996 [31,52]. *S*_*soc*_ thus requires further study.

Soil carbon pools could become a source or sink of CO_2_. Carbon emissions from soil to atmosphere would increase dramatically if SOC stocks decreased, which would intensify the greenhouse effect. The amount of carbon emitted from soil to the atmosphere globally is estimated at 40–50 Pg C annually, and the concentration of CO_2_ in the atmosphere would increase by 5, 12.5, and 20 mg·kg^-1^ worldwide if the content of soil organic matter decreased by 1, 2,and 3%, respectively [25,32,51]. The reverse ecological successions in our study area (FL-GL, FL-UL, GL-UL, SL-UL, GL-SL, and FL-SL) led to the loss of SOC, so the soil carbon pool would be a source of CO_2_. Positive successions (UL-GL, UL-FL, UL-SL, SL-GL, SL-FL, and GL-FL) would increase SOC stocks, indicating a CO_2_ sink. These results are similar to those of previous studies [16,47]. For maintaining the stability of the function of soil carbon pools, studies should therefore focus more on land-use/cover types in the region of the Tarim River; for stabilizing soil carbon storage to ensure the protection of desert riparian forests, more attention should be paid to maintaining the stability of vegetated areas and to the construction of species composition and community structure in arid areas. This study provides specific information on the effects of land-use/cover changes on SOC stocks in an arid area and offers regionally based, policy-relevant information on the impact of regional policy on the management of land use/cover.

### Conclusion

The area and structure of land use/cover changed during 2000–2010 along the main channel of the Tarim River. Specifically, they increased in CL, urban land, and SL but decreased in FL, GL, WB, and UL. SOC stocks in FL, GL, and UL were lower in 2010 than 2000. SOC content decreased with depth in all types of land use/cover except UL in 2010. Soil bulk density was lowest in the 0-5cm layer in all four types in 2010. *S*_*soc*_ in the 0–100 cm profiles was lower in FL, GL, and UL but higher in SL in 2010 than 2000. FL-SL, FL-GL, FL-UL, GL-SL, GL-UL, and SL-UL conversions led to the loss of *S*_*soc*_. *S*_*soc*_ increased for the opposite conversions in the sequence UL-SL>UL-FL>GL-FL>UL-GL>SL-FL≈SL-GL. Total *S*_*soc*_ in the four types of land use/cover was higher in 2010 than 2000. The improvement in SOC content depends upon an increase in the vegetation conversion (e.g. UL-FL), but it is also deeply related to the type of vegetation, the stability of vegetated areas, species composition and community structure.

## Supporting information

S1 Table Data for soil organic carbon content, etc. (XLSX)
